# Capturing Everyday Parental Feeding Practices and Eating Behaviors of 3- to 5-Year-Old Children With Avid Eating Behavior: Ecological Momentary Assessment Feasibility and Acceptability Study

**DOI:** 10.2196/66807

**Published:** 2025-02-27

**Authors:** Abigail Pickard, Katie Edwards, Claire Farrow, Emma Haycraft, Jacqueline Blissett

**Affiliations:** 1 School of Psychology and Institute of Health and Neurodevelopment, Aston University, Birmingham, United Kingdom; 2 Department of Clinical Psychology, School of Health in Social Science, University of Edinburgh, Wilkie Building, Edinburgh, United Kingdom, 44 1316501000; 3 School of Psychology, University of Birmingham, Birmingham, United Kingdom; 4 School of Sport, Exercise and Health Sciences, Loughborough University, Loughborough, United Kingdom

**Keywords:** pediatric, paediatric, child, child eating, parent feeding, parent, ecological momentary assessment, mHealth, mobile health, mobile app, application, smartphone, digital, digital health, digital technology, digital intervention

## Abstract

**Background:**

The wide use of smartphones offers large-scale opportunities for real-time data collection methods such as ecological momentary assessment (EMA) to assess how fluctuations in contextual and psychosocial factors influence parents’ feeding practices and feeding goals, particularly when feeding children with high food approaches.

**Objective:**

The main objectives of this study were to (1) assess parents/caregivers’ compliance with EMA procedures administered through a smartphone app and (2) estimate the criterion validity of the EMA to capture children’s eating occasions and parents’ feeding practices. Participant adherence, technological challenges, and data quality were used to provide an overview of the real-time dynamics of parental mood, feeding goals, and contextual factors during eating occasions.

**Methods:**

Parents in the United Kingdom with a child aged 3 to 5 years who exhibit avid eating behavior were invited to participate in a 10-day EMA study using a smartphone app. Of the 312 invited participants, 122 (39%) parents initiated the EMA study, of which 118 (96.7%) completed the full EMA period and the follow-up feasibility and acceptability survey.

**Results:**

Of those parents who completed the EMA study, 104 (87.4%) parents provided at least 7 “full” days of data (2 signal surveys and 1 event survey), despite 51 parents (43.2%) experiencing technical difficulties. The parents received notifications for morning surveys (69.9% response rate), 3 daily mood surveys (78.7% response rate), and an end-of-day survey (84.6% response rate) on each of the 10 days. Over the EMA period, a total of 2524 child eating/food request surveys were self-initiated by the participants on their smartphones, an average of 2.1 times per day per parent (SD 0.18; min=1.7, max=2.3). The majority of parents felt that the surveys made them more aware of their feelings (105/118, 89%) and activities (93/118, 79%). The frequency of daily food requests estimated by parents at baseline was significantly correlated with the frequency of food requests reported daily during the EMA period (*r*=0.483, *P*<.001). However, the number of daily food requests per day estimated at baseline (mean 4.5, SD 1.5) was significantly higher than the number of food requests reported per day during the EMA period (mean 3.7, SD 1.1), (*t*_116_=18.8, *P*<.001).

**Conclusions:**

This paper demonstrates the feasibility of employing EMA to investigate the intricate interplay between parental mood, feeding goals, contextual factors, and feeding practices with children exhibiting an avid eating behavior profile. However, the use of EMA needs to be carefully developed and tested with parents’ involvement to ensure successful data collection.

## Introduction

Children with an avid eating behavior profile pose unique challenges for parents, as this group of children has been shown to make greater requests for food, often in response to emotions and food cues in their environment rather than due to internal hunger signals [1,2]. Avid eating behavior in children is characterized by an intense and persistent interest in food, often leading to distinct challenges in managing feeding interactions [1,2]. Understanding the real-time dynamics of these interactions is crucial for developing targeted interventions aimed at promoting healthy eating behaviors in children.

However, despite evidence that both parental feeding practices and children’s eating behaviors are dynamic, much research has investigated the association between the 2 constructs cross-sectionally, using questionnaires, interviews, and one-off mealtime observations [3]. Such research only allows between-subject investigation, neglecting the within-subject variation in both feeding practices and eating behavior. These limitations have spurred more recent research using ecological momentary assessment (EMA; also known as experience sampling methods).

EMA is a form of mobile health technology that allows the collection of comprehensive data on daily life, as it is directly experienced by an individual, by asking them to provide responses about their emotions, context, and behavior at several random time points each day over several days [4]. Commonly, EMA employs both signal-contingent surveys, which are scheduled by the researcher to be push-notified at specific times, and event-contingent surveys, which are to be initiated by the participant following a specific event or action [5]. Due to advancements in technology and the widespread use of smartphones, EMA methods have become more popular in eating behavior research [4,6]. Berge et al [7] conducted an EMA study examining feeding practices in a sample of parents (N=150) of a 5‐7-year-old child with at least one other sibling between 2 and 12 years old. Their EMA study had very high compliance; 100% of participants (N=118) completed all 8 days of EMA data collection and met the minimum requirement per day (ie, 2 signal contingent, 1 event contingent, and 1 end-of-day contingent), with an average of 7.4 surveys per day [7].

A more recent study conducted by Loth et al [8] used EMA among a sample of 120 young adults with at least 1 child aged 2‐5 years. Of the 118 participants who registered for their EMA study, 116 (98.3%) participants completed at least one eating occasion survey, 100 participants completed one “full” day (84.7%), and 80 (67.8%) participants completed “full” days for the complete 10-day EMA period (a “full” day was defined as providing data for 2 eating occasions, 2 random prompts, and 1 end of day prompt). Finally, a study examining parents’ feeding practices as a predictor of children’s physical activity and fruit and vegetable intake used an EMA design with 22 mothers, fathers, and 8‐12-year-old-child triads completing a 7-day assessment period [9]. The response rates for the study period were high with 85%, 80%, and 85.9% responses to the survey notifications completed by the mothers, fathers, and children, respectively.

While these studies have provided important evidence for the use of EMA to investigate parental feeding interactions with children, there remains caution and concern within the field about the feasibility of using such methods with samples in which feeding and eating interactions may be more challenging. For example, it is unknown whether such methods are adequate to capture real-time information for parents of children with avid eating tendencies, who have previously been shown to have a higher risk of food insecurity [1] and where children are more likely to show an increased frequency of eating and food requests [2]. Therefore, this study assessed the feasibility, acceptability, and criterion validity of using EMA to explore the participation and everyday experiences of parenting children with avid eating behavior aged 3 to 5 years old across a 10-day sampling period.

Assessing the feasibility of such methods is not only important to guide future EMA research but also to ensure that the findings of this study are reliable. The study also aimed to determine the usefulness of using a commercially licensed software application on Android and iOS devices as a tool for EMA.

## Methods

### Overview

This EMA study was part of a larger program of research, the APPETItE project, which examines feeding and eating in preschool children with avid eating behavior to inform future intervention design and efficacy. An avid eating behavior profile in preschool children was identified and defined in an earlier APPETItE study using latent profile analysis (LPA) [1]. The LPA used the 8 subscales of the Children’s Eating Behavior Questionnaire (CEBQ) [10] as index variables to create holistic yet distinct eating profiles for UK children aged 3 to 5 years.

### Ethical Considerations

Recruitment, enrolment, and data collection for the EMA study took place between October 2023 and April 2024. Ethical approval was granted by the Aston University Health and Life Sciences Research Ethics Committee (HLS21079). All participants provided informed consent and were made aware that they had the right to opt out of the study at any time. For a comprehensive overview of the EMA study protocol and the measures included in each data collection wave, refer to Edwards et al [11]. The findings of the full study will be published at a later date.

### Participants

Only parents of children aged 3 to 5 years who were assigned to the avid eating profile based on the screening questionnaire were eligible to participate in this EMA study. Eligibility criteria also included English-speaking primary caregivers from the United Kingdom who were responsible for feeding their child for more than half the time when their child was at home. Parents whose child was autistic, or had severe learning disabilities, or a chronic illness that directly influenced their dietary requirements and eating habits were not eligible to participate. A total of 312 parents met the eligibility criteria for the study and were invited to participate via email.

### Recruitment

From the original LPA defining the avid eating profile, 101 parents with a child in the eligible age range (3‐5 y) and assigned to the avid eating profile were invited to participate. The profile solution identified in the LPA study [1] was used to classify additional children whose parents expressed interest in the EMA study and submitted CEBQ [10] data through the recruitment platform Prolific. Using this profiling method, 168 children were identified as belonging to the avid eating profile. A further 43 parents who had completed the CEBQ screening questionnaire for a separate lab-based study as part of the APPETItE project were also identified as having eligible children and were invited to take part in the EMA study. If the parents completed the initial interest survey, they were then contacted via email with further instructions and a demo video [12] explaining how to complete the EMA study. If parents experienced any technical difficulties, had any questions, or wanted to go through the study procedures, they were able to contact the research team by email.

### Data Collection Procedures

Participation in this study was remote; surveys were administered through a smartphone app, ExpiWell, which was downloaded directly to parents’ smartphones. ExpiWell is a cloud-based platform designed for researchers to conduct experience sampling methods and EMA studies. It enables researchers to create, schedule, and distribute surveys to participants via free mobile applications available on iOS and Android devices. Parents who did not own a smartphone were informed that they could request one from the research team to use for the study period, but all parents used their own devices.

Parents completed a baseline questionnaire, 10 days of EMA, and an end-of-study questionnaire. The 10-day EMA period included both signal surveys (push notifications sent to the participant’s smartphone) and event surveys (initiated by the participant following an eating occasion or request for food by the child). Parents were notified to complete 1 start-of-day survey, 3 signal-contingent examining mood and emotions, 1 end-of-day survey, and event surveys activated by the parent when their child ate or asked for food. Participants were able to schedule the morning and evening surveys to suit their typical routine. The full protocol and procedure are reported elsewhere [11].

Parents received a £100 (approximately US $126) shopping voucher if at least 8 “full” days of surveys were complete. Based on previous research, the criteria for one full day of EMA data included the completion of 2 signal-contingent surveys (morning, mood, or end-of-day) and one event-contingent (food) survey [8]. All parents who completed 10 days of EMA were entered into a prize draw to win an additional £100 (approximately US $126) shopping voucher. If participants withdrew prematurely from this study, their time was reimbursed on a pro-rated basis of £10 (approximately US $12.60) per complete day.

### Measures

To evaluate the feasibility and criterion validity of using EMA to capture the eating and feeding experiences of parents with a child displaying avid eating, several questions were incorporated into the survey period. The baseline survey asked parents to report on average how many times their child requested food, and ate food, both on weekdays and weekends. In the end-of-day surveys, parents were also asked to report how many times the child had eaten or requested food on that specific day. These data were collected to determine how many of the average eating and food request occasions were being reported in the EMA period.

### Feedback Questionnaire

A self-constructed feedback questionnaire, based on the items used by de Vries et al [13] and “The Open Handbook of Experience Sampling Methodology” [14], was used to gather participants’ perspectives on the EMA procedure. Participants responded to 16 items about their experience of completing the study, the influence the EMA study had on their daily lives, the awareness the EMA study gave them of their actions and feelings, and the burden of completing the EMA study. Response options were based on a 5-point scale where 1=“not at all,” 2=“a little,” 3=“moderately,” 4=“quite a bit,” and 5=“extremely.” The final item asked about whether the participant had experienced any technical problems during the EMA period with response options of 1=“yes,” and 0=“no.” The full study, including a list of all the items used, is available on the Open Science Framework [15].

### Data Analysis

Descriptives of the survey completion rates, forgotten reporting, withdrawal rates, nonresponse rates, survey duration, and any reported technical issues were examined to determine EMA feasibility. Criterion validity of EMA-reported frequency of eating occasions and food requests was examined by comparison with the initially reported frequencies and end-of-day frequencies using paired *t* tests and Pearson correlations. Study acceptability was assessed with descriptive statistics of the feedback responses at the end of the study.

## Results

### Demographics

Parents had a mean age of 34.97 years (SD 5.3; min=23.6, max=55.3) and children had a mean age of 52.57 months (SD 10.5; min=36.8, max=71.4). An overview of the parents’ demographic background and child sex is provided in Table 1.

**Table 1.** Demographic details of the sample (N=118).

Demographics	Values, n (%)	
Child sex		
Male	55 (46.6)	
Female	63 (53.4)	
Parent sex		
Male	26 (22)	
Female	92 (78)	
Parent ethnicity		
Asian	13 (11)	
Black	10 (8.5)	
White	93 (78.8)	
Other	2 (1.7)	
Education		
Degree	70 (59.3)	
No degree	48 (40.7)	
Parent or family structure		
Single parent	12 (10.2)	
Dual parent	96 (81.4)	
Cohabiting (not in relationship)	6 (5.1)	
Extended family	4 (3.4)	
Working status		
Unemployed	25 (21.2)	
Working part-time (between 8 and 29 hours per week)	36 (30.5)	
Working full-time (30 hours or more per week)	57 (48.3)	
Adequacy of income		
Living comfortably	54 (45.8)	
Managing	47 (39.8)	
Finding it difficult	17 (14.4)	
Household food security		
High or marginal food security	78 (66.1)	
Low food security	20 (16.9)	
Very low food security	20 (16.9)	

### EMA Feasibility

**Figure 1.**
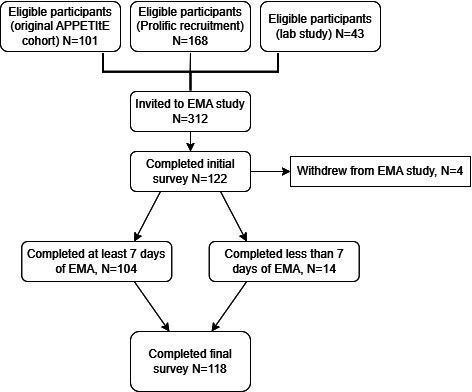
Recruitment and retention figures for each EMA phase. EMA: ecological momentary assessment.

Of the 312 parents invited to participate, 122 (39.1%) parents downloaded the mobile application and completed the initial survey. Of those 122 participants, 118 (96.7%) participants completed the EMA period. Four parents withdrew from the EMA study due to either technical difficulties or time constraints. Of the remaining participants, 104 (87.4%) participants completed at least 7 days of the EMA period with an average of 58 completed surveys for the full 10 days (SD 18; min=9, max=103). Figure 1 provides an overview of the eligible sample sizes as well as completion and attrition rates at each phase of the EMA study.

Over the 10-day EMA period, a total of 2524 child eating/food request surveys were self-initiated by the 118 participants on their smartphones, with 30 surveys partially completed. Parents self-initiated the request for food and eating surveys on average 2.1 times per day (SD 0.18; min=1.7, max=2.3). Of the 2524 eating and food request surveys completed at the moment, 2353 reported on an event in which the child ate food, and 171 reported on an instance in which the child requested food but did not eat.

At the end of all signal-contingent surveys and the food survey, parents were asked whether they had forgotten to report an eating occasion or a request for food from the child. On 870 occasions a parent retrospectively reported on an eating/food request that they had forgotten to report (Table 2 details the number of eating events that were reported on when the parent was reminded at the following survey).

**Table 2.** Number of retrospective eating occasions and food request reports.

Survey		Number of responses
Morning surveys		
Forgotten eating event		111
Forgotten food request event		4
Mood surveys		
Forgotten eating event		413
Forgotten food request event		28
End-of-day surveys		
Forgotten eating event		137
Forgotten food request event		13
Food surveys		
Forgotten eating event		141
Forgotten food request event		23
Total number of forgotten events reported		870

Response rates were calculated for the signal-contingent surveys, which included 1 survey in the morning, 3 surveys during the day, and 1 survey in the evening. From the first notification, parents had 60 minutes to complete the surveys before the link expired. A total of 1180 push notifications were received by all the participants (10 per participant) to complete the morning surveys, 825 morning surveys were initiated by participants following the notification, with 18 incomplete responses (response rate=69.9%). The parents also received notifications for 3 signal-contingent surveys (mood surveys) at semirandom times within one of three 120-minute blocks. A possible 3540 signal-contingent mood surveys were available to the 118 participants throughout the 10-day duration (30 per participant) with a total completion of 2778 surveys and 8 incomplete responses (78.7% response rate). The signal contingent end-of-day survey was sent to each participant every evening with a potential total of 1180 responses, 998 end-of-day surveys were initiated when prompted with 4 incomplete responses (84.6% response rate).

**Figure 2.**
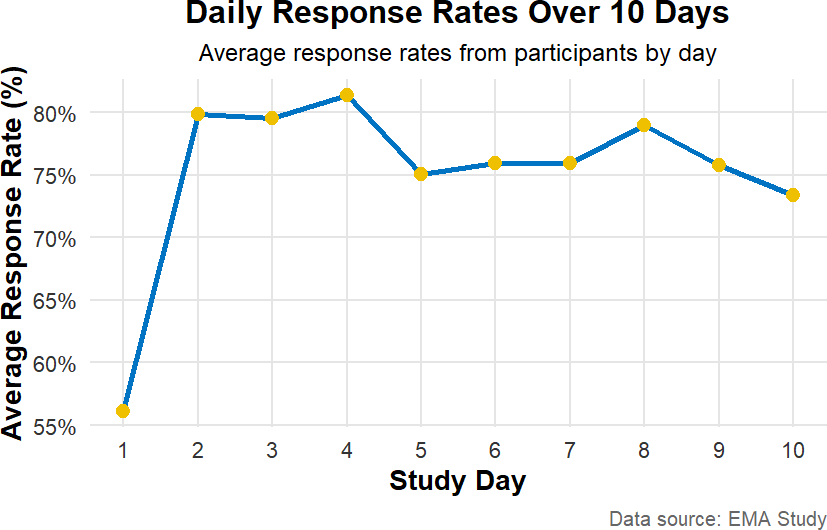
Response rates for signal contingent surveys over EMA Period. EMA: ecological momentary assessment.

To test whether more participants responded to the 5 prompted surveys at the beginning (eg, 2-4 d) compared with the end (eg, 8-10 d) a paired *t* test was conducted on the daily compliance rates. Day 1 was treated as a practice day due to significantly lower compliance rates as participants became accustomed to the EMA protocol (Figure 2). There was no significant difference in compliance rate for the start of the study period compared with the end of the study period (*t*_118_=0.127, *P*=.90).

A 1-way ANOVA was conducted to determine whether the working status of the parent was associated with the number of surveys completed. There was a significant difference in the number of reported eating and food request occasions (*F*_2,115_=12.41, *P*=.011), with parents working part-time reporting significantly more food occasions (mean=2.5 food surveys per day) than parents working full-time (mean=1.75 food surveys per day) but not significantly more than unemployed parents (mean=2.13). There were no significant differences in survey completion for all signal-contingent surveys between parents of differing working statuses.

Table 3 displays the descriptives of survey completion duration. The average duration to complete the morning surveys was 2.76 minutes (SD 6.83 minutes). The average time taken for the signaled mood surveys was 2.09 minutes (SD 6.16 minutes). The average duration to complete the food surveys was 3.13 minutes (SD 7.87 minutes) and 2524 food surveys were self-initiated and completed.

**Table 3.** Average survey completion duration by survey type.

	Duration (MM:SS)		
	Mean (SD)	Minimum	Maximum
Signaled surveys			
Morning surveys	02:29:00 (04:00:00)	00:38:00	58:32:00
Mood surveys	01:52:00 (02:21:00)	00:27:00	39:17:00
End-of-day surveys	02:20:00 (02:15:00)	00:47:00	31:44:00
Event surveys			
Food surveys	02:47:00 (02:29:00)	00:48:00	52:47:00

Almost half of the participants experienced technical issues with the application (51/118, 43.2%). Pearson chi-square test with Yates’ continuity correction revealed that Android users were more likely to experience technical issues (30.16% of iOS users experienced technical issues compared with 62.26% of Android users).

Over half of the participants (71/118, 60%) reported that they did not make any mistakes when completing the surveys.

### Criterion Validity

To determine the criterion validity of using EMA to accurately capture eating occasions and food requests of parents from children with avid eating, several additional measures of the frequency of children’s eating and requests for food were taken.

Parents were asked at baseline to estimate how frequently their child ate on a typical day, during the EMA period they were also asked to report how many times their child had eaten that day in the evening survey. A paired-samples *t* test and paired-samples correlations were then conducted to determine whether the scores were representative of what was reported by parents. Although the parents’ baseline reports of eating frequency were correlated with daily reported eating frequency (*r*=0.365, *P*<.001), eating frequency was significantly higher in the baseline reports compared with the daily reports (*t*_116_=5.78, *P*<.001). Parents reported greater eating frequency in the baseline survey (mean 4.5, SD 1.5) compared with the frequency that they reported in the end-of-day surveys (mean 3.7, SD 1.1).

Pairwise *t* testing showed that the approximate frequency of food requests per day reported at baseline was significantly correlated with the frequency of food requests reported daily during the EMA period (*r*=0.483, *P*<.001). However, pairwise *t* testing showed that the number of food requests per day at baseline was significantly higher (mean 5.6, SD 2.4) than the number of food requests reported per day during the EMA period (mean 1.9, SD 1.1; *t*_116_=18.8; and *P*<.001). The number of daily food requests and eating occasions reported by parents in the baseline survey was not significantly correlated with the number of daily food requests and eating occasions reported by parents in the EMA period (*r*=0.121, *P*=.097). Furthermore, the mean number of daily eating/request events at baseline (mean 10.1, SD 3.5) was significantly higher than the number of daily eating/request events during the EMA period (mean 2.04, SD 1.2; *t*_116_=24.9, and *P*=.001). The frequency of the daily eating/request events reported in the end-of-day survey was significantly correlated with the number of reports for in-the-moment eating/request events (*r*=0.244, *P*=.004). However, reports of average eating and food requests at the end of the day (mean 5.6, SD 1.8) were significantly higher than the average frequency of in-the-moment reporting of eating/food requests (mean 2.06, SD 1.2; *t*_116_=20.2; and *P*<.001).

### Study Acceptability

To determine whether the questions included in the EMA study were acceptable for measuring eating and feeding interactions with children with avid eating behavior, the parents were asked to rate the degree to which the questions included in the survey accurately reflected their experiences. The majority of the participants felt that the EMA surveys and questions accurately reflected their experience of a parent feeding a 3‐5-year-old child with avid eating behavior, 20% (n=24) said extremely well, 44% (n=52) said quite a bit, and 30% (n=35) said moderately well.

**Figure 3.**
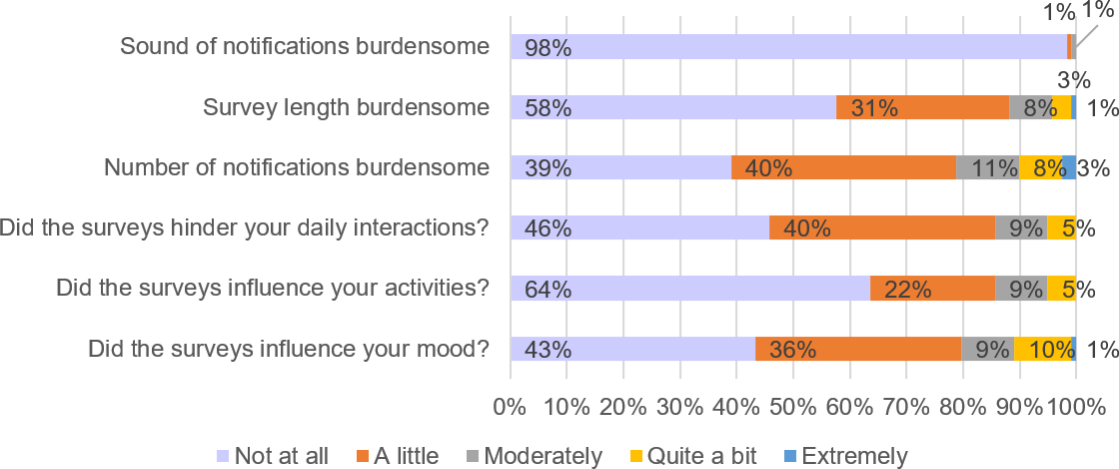
Proportions of responses for items measuring study acceptability.

Figure 3 presents participants’ feedback on the EMA period. Nearly all participants reported that they were not burdened by the sound of the study notifications (116/118, 98%). Although parents were asked to keep their devices on loud, it was at their discretion whether they muted the notifications. Over half of the participants were not burdened by the length of the surveys (68/118, 58%). Over half of the participants considered the number of notifications at least a “little” burdensome (73/118, 62%), but 39% (n=46) did not consider the frequency of notifications a burden. Over half of parents (64/118, 54%) found that the surveys hindered their daily interactions a little to quite a bit. However, (76/118, 64%) reported that the surveys did not influence their activities at all and (51/118, 43%) stated that the surveys did not influence their mood.

The majority of parents (105/118, 89%) reported that filling in the surveys made them more aware of their feelings and this was experienced as pleasant by 55% (58/105), neutral by 37% (39/105), and unpleasant by 8% (8/105) of parents. A total of 79% of parents (93/118) felt they were made more aware of their activities through completing the EMA study, of which 58 parents experienced this as pleasant, 34 parents experienced this as neutral, and 1 parent experienced this as unpleasant.

Parents were also asked in the signal-contingent end-of-day survey to rate how difficult it was to fill out the surveys that day, with 1=“not at all” and 5=“extremely”. After the scores had been aggregated by participants the mean level of difficulty was 2 (equivalent to “a little”) (SD 0.8; min=1, max=5).

## Discussion

### Principal Findings

This study aimed to assess the feasibility, acceptability, and criterion validity of using EMA to investigate parental feeding practices and their dynamic interactions with children exhibiting avid eating behaviors. The results demonstrate that EMA is a feasible and acceptable method for capturing real-time data on child eating occasions and food requests, but it also highlights some discrepancies in the data collected and usability issues specific to parents with young children.

### Feasibility of, and Compliance With, EMA Methodology

Despite the monetary incentive for the study, the recruitment rate to the study was lower than expected with only 122 (39.1%) of contacted parents initiating the study. However, the study found a high completion rate of 96.7% (n=118) for the EMA period, suggesting that the method was generally feasible for the participating parents. Most parents (104/118, 87.4%) completed at least 7 days of the 10-day EMA period, with an average of 58 surveys completed per participant over the 10 days, which is above the minimum requirement of 2 signal-contingent surveys and 1 event-contingent survey per day. The overall response rates for the signal-contingent surveys were relatively high, ranging from 69.9% to 84.6% depending on the type of survey. These findings suggest that it was generally feasible for the participants to engage with the EMA protocol, despite the demands of responding to notifications to complete signal-contingent surveys as well as self-initiating surveys multiple times per day. However, working status played a role in the frequency of reported eating and food requests, with part-time working parents reporting more food occasions than full-time working parents. This finding suggests that the employment status of parents may influence the ability to engage with EMA protocols, which could have implications for future studies in families.

The inclusion of pro-rated remuneration appeared to be valuable for retaining participants and appropriately rewarding ongoing completion of study components. A small percentage of parents withdrew from the study due to technical difficulties or time constraints, and nearly half of the participants (51/118, 43.2%) experienced technical issues with the application, which could have affected data quality and study adherence. The provision of an email address through which participants could access rapid technical support from our study team ensured the retention of most participants in the study despite these challenges. Such support must be built into any future studies using EMA methods to ensure participant retention. Careful testing and consideration of the most appropriate software are crucial in future EMA research. The feedback also indicated that while the majority of parents did not find the sound of notifications burdensome, some participants perceived the frequency and timing of surveys as a hindrance to daily activities. These findings highlight the importance of carefully considering the design and user experience of the EMA app to minimize the burden on participants and reduce barriers to accurate data collection.

### Criterion Validity of EMA for Capturing Feeding Interactions

While the EMA method showed promise in capturing in-the-moment data on eating occasions and food requests, discrepancies were found between the EMA reports and baseline recall data, suggesting that EMA should be validated with observational studies, given the challenges with any self-report methods. There were notable discrepancies between baseline reports of perceived frequency and EMA data of reported eating episodes: parents reported significantly fewer eating occasions and food requests during the EMA period compared with their baseline estimates. This pattern was consistent across different measures of eating frequency and food requests, suggesting a possible overestimation in baseline self-reports, or, perhaps more likely, underreporting of events during the EMA period. The finding that participants retrospectively reported missed eating or food request occasions in subsequent surveys supports the latter interpretation, suggesting some level of underreporting in the initial EMA responses.

There were 870 instances where parents, when prompted, reported missed eating or food request events that they had forgotten to log in the moment, highlighting a significant gap in real-time reporting accuracy. This retrospective reporting underscores the challenges inherent in capturing accurate eating behaviors in real-time, particularly with parents of young children. These results are consistent with previous studies indicating that retrospective prompts can improve data accuracy in EMAs [16]. The high incidence of missed reports emphasizes the importance of considering both real-time and reflective reporting methods in future research to enhance the validity and comprehensiveness of monitoring children’s eating behavior [16]. Thus, whilst EMA may provide a good assessment of some eating interactions demonstrated by the wealth of data we collected; it is unlikely to capture all such interactions in the period of study.

The observed discrepancies between baseline reports and EMA data reflect the challenges associated with self-reporting in real-time contexts, particularly for parents of young children who display avid eating. If parents are unable to complete surveys at the moment due to competing demands or distractions, the more chaotic or stressful feeding interactions that we aim to capture are likely to be missed. Additionally, the high frequency of child eating behaviors and food requests, particularly among children with avid eating behaviors, may lead to reluctance in parents to consistently report these events [2]. Despite these limitations, the general correlation between EMA-reported data and baseline data indicates that EMA remains a valuable tool for capturing dynamic changes in feeding practices and children's eating behaviors. However, to mitigate these challenges, future EMA studies could incorporate design modifications, such as reducing the EMA period, providing more frequent reminders, or simplifying survey interfaces to encourage immediate and consistent reporting.

### Acceptability of EMA

The study’s findings indicate that our EMA protocol was generally acceptable to parents. The majority of participants reported that they were not burdened by the notification sounds (116/118, 98%) and were not significantly affected by the length of the surveys (68/118, 58%). However, the frequency of notifications was considered burdensome by some participants (73/118, 62%), and over half (64/118, 54%) reported that the surveys hindered their daily interactions to some extent. Despite this, most parents reported that completing the EMA surveys increased their awareness of their feelings and activities, which was perceived as pleasant or neutral by the majority. These findings suggest that while EMA can be intrusive for some participants, it is generally well-tolerated and may even have positive side effects by enhancing self-awareness.

### Implications for Future Research

The findings of this study have important implications for future research using EMA to investigate feeding practices and eating behaviors in children. First, the study underscores the need to further refine the EMA methodology to ensure that it is user-friendly and minimizes the burden on participants. For example, the user interface could be enhanced to reduce technical difficulties, and the frequency and timing of surveys could be further optimized based on participant feedback.

Second, the study provides evidence supporting the use of EMA to capture in-the-moment mood and context data related to parental feeding practices and children’s eating behaviors. This is particularly important given the dynamic and context-dependent nature of these behaviors, which cannot be adequately captured through traditional cross-sectional methods. By employing EMA, researchers can gain a more nuanced understanding of how contextual factors, such as parental mood, feeding goals, and external pressures, influence feeding practices on a moment-to-moment basis.

Finally, this study contributes to the growing body of literature supporting the use of mobile technology for real-time data collection in behavioral research. The high compliance rates observed in this study suggest that parents are willing and able to use smartphone apps to report on their feeding practices and their children’s eating behaviors, even over extended periods. This opens up new opportunities for large-scale studies that leverage mobile technology to explore complex behavioral dynamics in diverse populations.

### Limitations

It is important to recognize that EMA methods have certain limitations. Since assessments are conducted repeatedly and in close relation to the behaviors, thoughts, and emotions being studied, there is a chance that the methods themselves could influence the variables being examined. This indeed is evidenced in the high percentage of participants who said they were made more aware of their emotions and activities because of the study. As such, there is the potential that throughout the EMA period, an individual’s behaviors and self-reports begin to shift and this should be accounted for in subsequent analysis. Future studies should incorporate features to address participant reactivity, such as randomizing the order of questions.

It must also be noted that there are several general limitations with EMA methodologies. In particular, the surveys are self-reported so this may result in the same social desirability reporting that is seen in other self-report methods such as unrepeated questionnaires. Additionally, in an attempt to keep the surveys brief and accessible, very basic responses (ie, yes or no) were provided, which limits capturing the detail and nuance of eating occasions. Ideally, examples of child-parent interactions should be observationally recorded to allow for a richer interpretation of feeding interactions in young children with avid eating as well as determining the accuracy of parent reports. The results of this study also highlight that relying on parents to trigger event-based surveys often results in underreporting of eating occasions which impacts the accurate reporting of the frequency of eating occasions or food requests in children with avid eating. The development of wearable devices and camera recordings could be used in future research to capture more precise data on children’s eating patterns.

Additionally, while EMA studies can be designed to include a diverse sample with various racial, ethnic, and socioeconomic backgrounds, it is essential to consider how likely different groups are to complete an EMA survey and how well the results can be generalized to the broader population. Although participants were offered the use of a smartphone for the study, no participant requested this, but it may still be the case that access to a smartphone was a barrier to participation. Equally, participants with higher completion rates than others may have greater time and fewer pressures around feeding interactions to be able to complete the surveys. Furthermore, there is also the possibility that parents who choose to participate and complete an EMA study may have a stronger interest in promoting healthy eating habits for their child than those who choose not to participate, which could introduce bias into the findings. Finally, although the adherence in our study aligns closely with previously reported short-term EMA studies [17], the 10-day duration may limit the ability to generalize to contexts requiring sustained monitoring over longer periods.

### Conclusion

In conclusion, this study demonstrates the feasibility, acceptability, and potential of using EMA to investigate the intricate interplay between parental mood, feeding goals, contextual factors, and feeding practices in children with avid eating behaviors. While the results are promising, further refinement of the EMA methodology is needed to address technical challenges and reduce participant burden. Future research should continue to explore the use of EMA to capture the dynamic nature of feeding interactions and to inform the development of targeted interventions to promote healthy eating behaviors in children.

## Abbreviations

CEBQ: Children’s Eating Behavior Questionnaire
EMA: ecological momentary assessment
LPA: latent profile analysis
